# Supplementary material for: Feasibility of using a novel automatic cardiac segmentation algorithm in the clinical routine of lung cancer patients
Source: PLoS One. 2021 Jan 14;16(1):e0245364. doi: 10.1371/journal.pone.0245364 (PMC7808597; doi:10.1371/journal.pone.0245364)
Supplement: Attachment — Submitted filename: Reviewer responses.docx [file pone.0245364.s001.docx]

Reviewer responses

We would like to extend our sincere thanks to the reviewers for providing constructive comments and thoughtful suggestions, which we feel have contributed to an improved manuscript. Overall, this has produced a more detailed and thorough description of both our work and the clinical and research context in which it is placed. Below we address each comment individually.

Reviewer 1

Comment 1. The major question of this study is that how the proposed method working in clinical practice? How about the reproducibility? The various image quality might be a problem for the proposed method.

Response 1. Thank you for this question. We agree that this is a primary concern. We have provided additional details regarding how we will implement our method in clinical practice, and discuss the challenge faced with variable image quality (Discussion - paragraph 3).

Comment 2. The literature review of the work is highly insufficient, and the authors missed the most recent deep learning based segmentation methods. The authors should cite the following publications with proper discussions. (citations follow)

Response 2. We greatly appreciate the feedback and have taken this into consideration. We agree that many recent advanced in deep learning show promise for translation into the clinic, and have discussed the suggested published articles, along with several more, in detail in the revised manuscript (Discussion - paragraphs 6 and 7).

Comment 3. Ideally, the authors should consider to compare with some deep learning based methods or at least have some discussions.

Response 3. Thank you again for this suggestion. Following from comment 2, we have also provided a comparison to recent relevant research for cardiac delineation.

Comment 4. Why the automated results represent lots of ragged artefacts?

Response 4. Thank you for this question. Using a multi-atlas approach, the automatic segmentation is essentially spatially averaged in 3D. Since tomographic imaging is reconstructed in axial planes, these segmentations can look jagged when viewed in 2D slices as a results of these factors. In three dimensions, automatic segmentations often look much smoother (as shown in Figure 2), which was a point raised by reviewer 2.

Reviewer 2

Comment 1. The auto-contouring method was used only for contouring on the zero-phase and propagation to other phases was done via deformable image registration. This method of contour propagation is acceptable and widely performed in RT, however since the authors are comparing the proposed method to manually contoured structures (contoured on each phase), this leads to a biased comparison. Furthermore, the authors are essentially testing the feasibility of using 4DCT and DIR to propagate contours (which has been widely published on) rather than using the automated segmentation framework. The proposed method should either be applied independently to each breathing phase or the manual contours on the 0% phase should be propagated via DIR to the other phases. Since deformation vector fields must typically satisfy a smoothness constraint, this may also explain why contours generated via the atlas-based and DIR method are smoother than manual contours.

Response 1. We greatly appreciate this in depth comment and the suggestion to further validate our methodology. In response, we have applied our automatic segmentation software to each phase independently. Results are given in Figures 3 and 4, with additional description in the revised manuscript (methods - final paragraph, results - final 2 paragraphs, discussion - end of paragraph 2).

Comment 2. Many recent studies have posited that dose to the whole heart may not be adequate for correlating radiation dose with cardiac toxicity outcomes [reference list]. Given that previous publications by the authors have shown that the auto-segmentation method can be applied to cardiac substructures, in addition to the whole heart, I would suggest extending the analysis in the manuscript to include contouring of cardiac substructures.

Response 2. Thank you for this suggestion. Firstly, we agree that much more research is required to better understand the link between radiation dose to cardiac substructures and cardiotoxicities. This is an area of research we are actively involved with (see our recent work published in Radiother. And Oncol.: <https://doi.org/10.1016/j.radonc.2020.09.004>).

The aim of this work was to demonstrate the feasibility of clinical implementation, and as current clinical practice does not consider cardiac substructures, we decided this extra information was not relevant for this study. With better cardiotoxicity models utilising cardiac substructure doses, it is likely this will change in the future, and we hope our tool will be able to be used to its full potential. Outside of clinical radiotherapy, this software might be applied for retrospective analysis of cardiac substructure doses more widely, as it has been already. We have discussed these points in the revised manuscript (Discussion - paragraph 5)

Comment 3. The manuscript is very brief, and the immediate impact of the proposed method in the clinic is not clearly highlighted. Additionally, visual results from only one or two patients are shown. It would be helpful to include the segmentation results of all 10 phases on one or two patient slices.

Response 3. Thank you for this suggestion. We believe that the revised manuscript presents a more complete analysis, and a more thorough investigation. Following your suggestion, we have included visual results for the best, median, and worst patient segmentation results (Figure 5).

Minor comments/concerns.

Comment 1. While some deep learning methods for automatic segmentation require large datasets, the method published by Morris et al required only 25 patient datasets for training, so the authors' claim may be unfounded.

Response 1. We thank you for bringing this to our attention. In the revised manuscript, we have added a description of deep learning methods and a comparison between relevant studies and our work (Discussion - paragraphs 6 and 7). It is correct that the work by Morris et al required only 25 cases to train a network, however a direct comparison to our framework is difficult as the data used by their group comprised paired CT/MRI (which is typically not available). Despite this, we are excited by the feasibility of using smaller datasets to train deep learning models, and look forward to future work in this area, as typically most studies present training datasets comprising much numbers of patient images.

Comment 2. As the authors mention in the discussion - large datasets may need to be used to reach definitive conclusions on the dosimetric impact of the proposed method, some study on the dosimetric impact should be included. A comparison in heart DVH based on contours from the proposed method, and manual contours on the MIP and average CT would be helpful.

Response 2. We appreciate this suggestion. In the revised manuscript we have included a dosimetric investigation for a patient selected from our study dataset with the smallest heart-tumour separation. We present a qualitative analysis of the differences in dose (see Figure 6), which we discuss (Discussion - paragraph 4).

Comment 3. The cardiac contours are propagated via respiratory-correlated 4DCT, which may not be associated with or fully capture cardiac motion. Given this is standard practice in radiotherapy clinics, it is not a major concern, but the authors should at least mention this in the discussion section.

Response 3. Thank you for this astute observation. We have raised this point in our revised manuscript (Discussion - end of paragraph 8)
